# Adult congenital heart diseases: systematic review/meta-analysis

**DOI:** 10.1097/MS9.0000000000001704

**Published:** 2024-01-11

**Authors:** Ahmdelmukashfi M.Elmustfa A.Azem Mahmod, Shima Hassan Mohammed Koko

**Affiliations:** a Faculty of Medicine and Health Sciences, Red Sea University, Port Sudan; b Faculty of Pharmacy, University of Khartoum, Khartoum, Sudan

**Keywords:** Adults, congenital heart diseases, quality of life

## Abstract

**Background:**

Congenital heart disease (CHD) is a gross structural abnormality of the heart that has functional significance. The impact of CHD on the patients’ quality of life (QOL) is a topic of considerable interest and importance to both researchers and clinician. However, there is a lack of systematic reviews investigating and identifying the QOL of congenital heart disease patients.

**Aim:**

To assess the QOL of adult patients with congenital heart disease by reviewing the previous studies conducted on this subject.

**Methods:**

The PubMed and Google Scholar databases were explored for studies published between 2020 and 2022. The keywords used for the searching process included “QOL, Adults, CHD, Outcomes, Impact, Effects, Life of CHD Patients.” The inclusion criteria were original English articles and full-text articles conducted on adult patients with congenital heart disease and reported quality of life.

**Results:**

A total of 5455 articles were obtained, but only seven articles were eligible for the inclusion criteria. The included studies involved a total of 8549 participants; 104 were healthy, and 8445 were adult patients with congenital heart disease. The investigated items of the studies included quality of life, health-related QOL, including physical and psychological dimensions, sense of coherence, mental health, physical functioning, physiological wellbeing, psychological resilience, anxiety, depression, illness perception, and health status.

**Conclusion:**

Patients with congenital heart disease experience low QOL across all dimensions. The risk factors for poor QOL included age, depression, anxiety, and female gender.

## Introduction

Highlights

- A systematic review assessed the quality of life of adult patients with congenital heart disease.
- Searching in databases was done between 2020 and 2022.
- Low quality of life was found in patients with congenital heart disease.

Congenital heart disease is a gross structural abnormality of the great intrathoracic vessels or the heart with actual or potential functional significance^1^. The total birth prevalence of congenital heart disease (CHD) is estimated to be 8 cases per 1000 births, ranging from 3 cases per 1000 for moderate or severe lesions to 13 cases per 1000 for milder lesions^2^.

CHD was considered a paediatric disease, as patients with moderate or severe disease complexity rarely survived beyond childhood^3^. In a historical cohort of CHD, almost 60% of infants born with CHD died during the first year^4^. Recent advances in diagnosis and management have transformed the prospects for these patients^5^. Therefore, more than 90% of CHD patients survive into adulthood, with a prevalence of adult congenital heart diseases (ACHD) estimated to be 3 per 1000 adults^6^. A study from Canada showed that the prevalence of ACHD among adults older than 65 years was 3.7 cases per 1000 indexed to the general population and was mainly in patients with valvular lesions and shunts^7^. Despite the progression in diagnosis, use of devices, percutaneous interventions, and improved survival, most patients are not cured. Many of them have residual problems of ongoing morbidity, which may lead to premature death^5^.

Traditionally, CHD outcomes were based on measuring functional status and mortality; however, there is an increasing recognition that reliance on clinical measures in determining outcomes isn’t sufficient as it fails to capture patients’ perspectives^8^. As a result, there is an increased interest in evaluating the quality of life (QOL) of adult patients with CHD^9^. Indeed, researchers and clinicians were intrigued by how the lives of CHD patients looked and whether heart defects influenced QOL^10^.

QOL does not have a globally accepted definition; however, it is usefully conceptualized in domains such as the physical, environmental, and psychological domains^9^. However, there is a lack of systematic analysis conducted to investigate and identify the QOL of CHD patients. The previous systematic analysis was published in 2013 and involved studies published earlier^9^. Despite a substantial increase in the published data on the QOL topic since 2020, there is an absence of recent systematic analyses on this subject. Therefore, this systemic review aims to provide a comprehensive understanding of the various domains of the QOL of adult patients with congenital heart disease to identify potential areas for improvement in their care. By analyzing previous studies, this review will contribute to the existing body of knowledge and facilitate the development of targeted interventions to enhance the QOL of these patients. Ultimately, the findings of this review will help healthcare professionals provide better support and guidance to adult patients with congenital heart disease, thereby improving their overall well-being.

### Method and search strategy

This systematic review has been reported in line with the PRISMA criteria and fully compliant with the PRISMA 2020 statement

#### Reporting section for the PRISMA statement

We adopted the PRISMA checklist guidance for systematic review and meta-analysis^11^ while writing this systematic review. Also, this study was reported in accordance with AMSTAR 2 (Assessing the methodological quality of systematic reviews) Guidelines^12^.

#### Electronic searches

Two scientific databases were used for searching scientific articles, including PubMed and Google scholar databases. These two databases are known for their inclusivity, which includes an extensive and diverse range of data sources, ensuring a comprehensive review of the research question. The authors acknowledge that other databases could contribute valuable insights. However, the decision was made based on practical considerations, including accessibility and relevance to the study’s objectives.

Several keywords were used for searching, including “QOL, Adults, CHD, outcomes, impact, effects, life of CHD patients.” All the titles produced for this exploration were reviewed, and all articles unrelated to the current subject, such as discussing other heart diseases, were excluded.

#### Eligibility criteria

After reviewing the titles of articles and including articles focused on congenital heart disease, the first step was to include articles conducted on the adult population and exclude articles conducted on the paediatric population. Also, we had articles published between 2020 and 2022 and excluded all articles published before 2020. This is because there has been a considerable increase in published data on the QOL of adult patients with CHD since 2020. Only articles in English were defined as articles of relevance, which were then included in the second stage.

**Figure 1:**
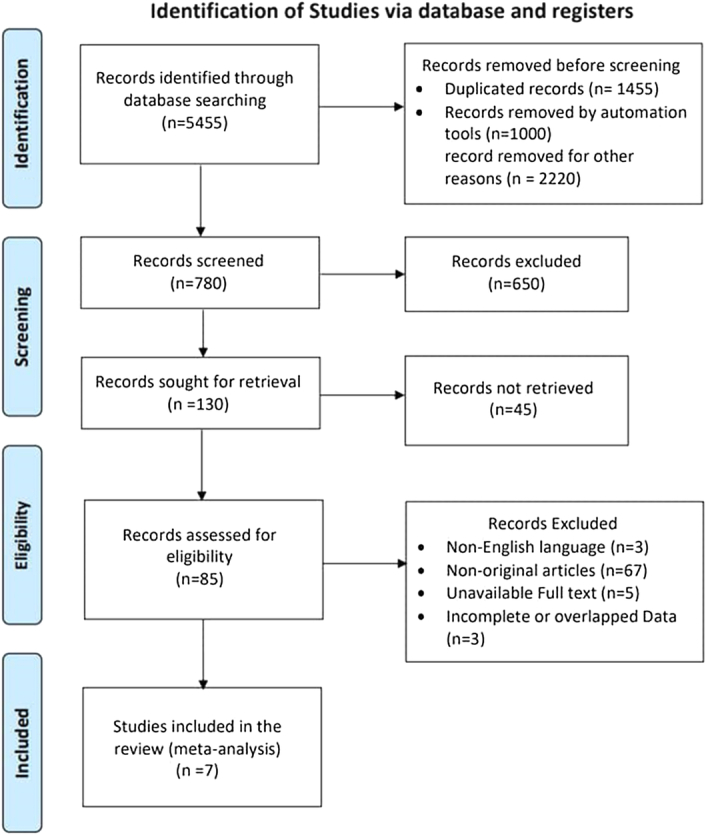
Prisma flow diagram.

The second step involved reviewing the abstracts of articles to include original articles and excluding other types of articles such as review articles, systematic reviews, letters to the editor, and case reports. The remaining findings were original articles written in English, conducted on adults, and published recently. The remaining articles were reviewed in full text to exclude studies with overlapped or incomplete data and unavailable full-text articles. The full description of the search strategy is shown in Fig. 1.

### Quality assessment

The JOANNA Briggs Institute (JBI) quality appraisal checklist was used in assessing the quality of included studies^13^. The number of items the JBI tool consisted of for cross-sectional studies was eight. The first is whether inclusion criteria are clearly defined. The second is appropriateness in the description of the study subject and setting. The third item is whether the measurement of exposure is valid and reliable. The fourth is the relevance of describing the objective and standard criteria used. Fifth is representing the identification of confounders appropriately. Sixth is the appropriateness of a strategy to handle confounders. The seventh is the reliability and validity of outcome measurement. Finally, the eighth one is the appropriateness of the statistical analysis method used. Studies with results of 50% and above are considered low-risk and were included in the analysis.

### Data review and analysis

The articles were reviewed to determine the main data that would be collected, such as study design, population information, the items investigated, results, and main findings. Then the full articles were reviewed, and the determined data were extracted into an Excel sheet for revising. After the revision of the collected data, the data was then transferred into a pre-designed table to summarize the findings.

## Results

### Characteristic of included studies

Seven studies were included in our analysis as they met the inclusion criteria^14–20^ (Table 1). All studies were cross-sectional; the total number of participants was 8549, and 104 were healthy individuals^15^. The populations in the studies included a congenital heart disease population of four diagnostic groups involving simple, tetralogy of fallot, transposition of the great arteries, and single ventricle^14^. Other studies included grown-up congenital heart patients^15^, congenital heart disease patients from 15 countries^16^, hospitalized congenital heart disease patients^17^, patients with different subtypes of congenital heart disease from 15 countries^18^, congenital heart disease patients classified according to anatomical and physiological classification^19^, and congenital heart disease patients. Two studies did not report the ages of patients^16,19^, another study reported the median age as 32 years^18^, and the remaining studies reported an age range of 16–76 years^14^.

**Table 1:** Characteristics and summary of included studies

Author and publication year	Study design	Population	Tool for measuring quality of life	Items investigated	Results and main findings	Quality
Fteropoulli *et al.* 2022^14^	Cross-sectional	*N*=303 (Simple, Tetralogy of Fallot, Transposition of the Great Arteries, Single ventricle) Age:18–76 years	Health-related quality of life (HRQoL)	Health-related quality of life *Physical *Psychological	*Comparing the generic and disease-specific HRQoL of the four diagnostic groups revealed that the Tetralogy of Fallot group had a higher physical component summary score than the general population, whereas the Simple group had a lower psychosocial HRQoL. *As a result of cardiac surveillance, the Single Ventricle group reported a lower HRQoL than the Simple group (mean difference, -5.99; *P* =0.003). *Major risk factors involve younger age, self-deprecation, anxiety, and depression.	Low risk
Chow 2021^15^	Cross-sectional	*N*=208 (104 health peers and 104 patients with congenital heart) Age:18–70 years	Satisfaction with Life Scale (SWLS)	SWLS Psychological resilience Anxiety Depression Illness perception	* Patients with CHD have lower QOL scores and health status than healthy peers, which are linked to greater disease severity and worse functional class (all *P* values<0.01). *SWLS and health status positively correlate with psychological resilience but negatively with hospital anxiety and depression scales and negative illness perception (all *P* values<0.01). *Reduced QOL and health status among grown-up CHD patients are associated with both clinical and psychological attributes.	Low risk
Moons *et al.* 2021^16^	Cross-sectional	*N*=4028 participants Population: Congenital heart disease patients from 15 countries Age:-------	Linear analogue scale (LAS)	Quality of life Sense of coherence	* The mean SOC score for patients with CHD was 65.5±13.2 and the mean QoL was 78.3 (±16.6), with lower scores associated with younger age, lower education, job seeking, disability, unmarried status, poor functional class, and simple CHD. SOC was positively associated with QoL in the total sample, with significant variation across countries.	Low risk
Truong *et al.* 2021^17^	Cross-sectional	*N*=109 adults with congenital heart disease patients hospitalized Age:16–>61 years	EuroQOL-5 dimensions-5 level (EQ-5D-5L)	Quality of life Health status Anxiety Depression	*A study found that 9.2% of patients with CHD experienced life dissatisfaction, with 18.7% experiencing anxiety and 11% experiencing depression. Patients aged over 30 years had lower QOL scores. * Stratified multivariate logistic regression revealed that poor QOL was linked to unemployment/unstable employment(OR 4.43, *P*=0.002), life dissatisfaction with unmarried status (OR 4.63, *P*=0.026), anxiety and depression related to unemployment/unstable employment (*P*<0.05) and complex CHD/PAH (OR 4.84, *P*=0.016). *Hospitalized adults with congenital heart disease often experienced reduced QOL and elevated psychological problems.	Low risk
Monns *et al.* 2021^18^	Cross-sectional	*N*=3538 participants Population: Patients with different subtypes of congenital heart disease from 15 countries Age: median 32 years	Linear Analog Scale (LAS)	Quality of life Mental health Physical functioning	* Patients with coarctation of the aorta and isolated aortic valve disease reported better physical functioning, mental health, and QoL, while those with cyanotic heart disease or Eisenmenger syndrome had worse outcomes. *Some types of CHD predicted worse outcomes. However, it appears that it is the functional status associated with the heart defect rather than the heart defect itself that shapes the outcomes	Low risk
Martinez-Quintana *et al.* 2021^19^	Cross-sectional	*N*=191 patients with congenital heart disease patients classified according to anatomical and physiological classification	WHOQoL-Bref instrument	Quality of life	* The study analyzed the classification of CHD patients based on anatomical and physiological features. The study found no significant differences in the anatomical classification and QOL questionnaire sections between CHD patients, with a range of mild 23%, moderate 60%, and great defects 17%. *The study found that 36% of patients were in physiological Stage A, 14% in Stage B, 44% in Stage C, and 6% in Stage D, with patients in Stages C and D having significantly lower physical domain. However, no significant difference was found in the psychological, social relationships, or environmental domains. *The study also found that higher educational level was a protective factor [OR 0.32, *P*=0.026], while being married or cohabit was a risk factor for worse rated QoL [OR 3.46, *P*=0.030]. *Furthermore, having a worse functional class (NYHA ≥2) was associated with dissatisfaction with health [OR 3.44, *P*=0.021].	Low risk
Lopez Barreda *et al.* 2020^20^	Cross-sectional	*N*=68 participants with congenital heart disease patients Age:18–72 years	36-Item Short Form Health Survey (SF-36)	-Quality of life -Physiological wellbeing	*CHD patients reported worse scores in physical dimensions of quality of life, such as physical function (70.5), role functioning (64), vitality (65.3), and general quality of life(58.6), compared to emotional or social dimensions. *Female gender was associated with lower scores in physical function (59.12 versus 82.66; *P*<0.01) and role functioning (53.68 versus 75; *P*<0.05), while poverty was associated with worse results in physical function, role physical, vitality, social role functioning, and bodily pain (all *P* values were <0.05). *Psychological scales had an association with quality of life, but clinical variables did not show significant correlations.	Low risk

CHD, congenital heart disease; OR, odds ratio; PAH, pulmonary arterial hypertension; SOC, sense of coherence.

### Quality of life

The investigated items of the studies included QOL^19^, health-related quality of life including physical and psychological dimensions^14^, quality of life, and sense of coherence^16^, quality of life, mental health, physical functioning^18^, QOL and physiological wellbeing^20^, quality of life, psychological resilience, anxiety, depression, and illness perception^15^, and quality of life, health status, anxiety and depression^17^.

The studies reported different dimensions of quality of life; therefore, there were variations in the reported dimensions and, as a result, variations in findings. One study compared the QOL between CHD patients and their healthy peers; the study showed that patients had lower QOL and health status compared to healthy individuals^15^. Another study showed that 9.2% of patients experienced life dissatisfaction, with a prevalence of anxiety and depression of 18.7% and 11%, respectively^17^. CHD patients showed worse scores in general QOL and QOL regarding physical dimensions such as physical function, physical role functioning, and vitality than emotional and social dimensions^20^.

Regarding subgroups of CHD patients, the tetralogy of the Fallot group had higher scores compared to the general population regarding physical components. In contrast, the simple group showed a diminished psychological health-related quality of life compared to the general population. The comparison between the subgroups of CHD showed that the single ventricle group showed a poorer health-related quality of life than the simple group^14^. Patients with coarctation of the aorta and those with isolated aortic valve disease showed the best QOL, physical functioning, and mental health. On the other hand, patients with cyanotic heart disease or Eisenmenger syndrome had the worst outcomes^18^.

Regarding anatomical and physiological classifications of CHD patients, different anatomical classifications showed no significant difference in QOL aspects, including physical, psychological, social relationships, and environmental dimensions. In terms of physiological classification, patients with higher stages (C&D) of CHD had significantly lower physical domain scores than patients with stage A, with no significant variation regarding psychological, environmental, and social relationship domains^19^. ccQOL was found to, in turn, significantly and positively affect the sense of coherence of CHD patients^16^.

Factors affecting the QOL were reported, and risk factors for poor health-related QOL (psychological dimension) included young age, depression, anxiety, and self-blame^14^. Another study showed that QOL among patients older than 30 years was lower than those aged 30 and younger. Also, the following outcomes were found to be associated with each other: poor QOL with unemployment/unstable employment [odds ratio (OR)=4.43, *P*=0.002], life satisfaction with unmarried status (OR=4.63, *P*=0.026), anxiety with unemployment/unstable employment (OR=3.88, *P*=0.017) and complex CHD/pulmonary arterial hypertension, depression with unemployment/unstable employment (OR=4.63, *P*=0.003)^17^.

QOL and health status were significantly and positively associated with psychological resilience and negatively with hospital anxiety, depression, and negative illness perception (*P*<0.001). Moreover, predictors of QOL included lower resilience, great neuroticism, and illness perception (*P*<0.05)^15^. High education level was a protective factor for worse QOL (OR=0.32, *P*=0.026), whereas being married or cohabiting was a risk factor for worse QOL (OR=3.46, *P*=0.03)^19^. The female sex was associated with a lower physical function score (*P*<0.01) and physical role functioning (*P*<0.05). Poverty was also associated with worse results in QOL regarding physical function (*P*<0.01), role physical (*P*<0.01), vitality (*P*<0.05), social role functioning (*P*<0.05), and bodily pain (*P*<0.05)^20^. One study did not report any factors associated with QOL levels^18^.

## Discussion

Traditionally, the outcome of CHD has been estimated and measured in terms of functional status and mortality. However, reliance on clinical measurements only for the determination of the outcomes of CHD patients is not sufficient^9^. Therefore, it is necessary to investigate the QOL; therefore, we conducted this systematic analysis.

QOL involves a range of life domains such as role functioning, social relationships, environmental aspects, engagement in daily activities, mental health functioning, and physical abilities^21^. The diverse findings across studies regarding QOL domains indicate the complexity of assessing QOL in this population. This highlights the need for a comprehensive and standardized approach to QOL assessment within this demographic.

Research suggested that adult patients with CHD are similar to patients with chronic disease, and they are facing physical health issues and psychological challenges^22^. In our analysis, CHD patients showed lower QOL and health status compared to their healthy peers^15^. Simliarly, CHD patients exhibited worse scores in general QOL^20^. The comparison between these subgroups showed that the single ventricle group showed a poorer health-related quality of life compared to the simple group^14^.

Additionally, It was reported that patients with complex CHD, Eisenmenger syndrome, and the Fontan circulation have worse long-term survival rates^23^. This was in agreement with our findings; one study in our analysis showed that patients with cyanotic heart disease or Eisenmenger syndrome had the worst outcomes regarding QOL^18^. These findings could be attributed to the burden of managing a chronic condition, potential limitations in physical activities, and the emotional impact of living with a congenital heart condition, affecting both the subjective and objective overall well-being of CHD patients. Lower QOL and health status in adult CHD patients require holistic care approaches, including comperhencive counselling sessions.

The range of CHD defects varies from a single simple defect without symptoms to multiple complexes of defects with several symptoms^2^. In our analysis, significant variations were observed among subgroups of CHD patients, particularly in the physical domain. Patients with tetralogy of the Fallot^14^, also patients with coarctation of the aorta and isolated aortic valve disease demonstrating the best physical functioning, and mental health^18^. In contrast, patients with cyanotic heart disease or Eisenmenger syndrome experienced the worst outcomes^18^. This analysis also revealed that CHD patients, as a whole, exhibited lower scores in physical dimensions compared to emotional and social dimensions^20^. Further research is essential to understand the specific factors contributing to variations in the physical well-being of CHD patients across subgroups, guiding the development of targeted and effective care strategies.

In terms of physiological classification, the simple group exhibited diminished psychological health-related quality of life compared to the general population^14^ It was noted that psychiatric diseases have a negative impact on the QOL, pointing to the importance of measuring psychological factors such as mental disorders for the interpretation of QOL^21^. A large cross-sectional study with more than 20 000 participants showing lower QOL and increased sick leave with most mental disorders^24^. Psychological QOL and health status were potentially and negatively associated by the presence of mood, anxiety disorders^15,17,21^, and depression^15,17^. A study with 150 adults with chronic heart disease (CHD) found higher comorbidity rates with mood and anxiety disorders, with poor QOL observed in patients with anxiety, mood, or substance use disorders compared to those without any mental disorders^24^. However, comparisons between the findings of these studies have been hampered as some studies used multivariate analysis whereas other studies reported univariate analysis only^25^.

However, patients with higher stages (C&D) of CHD demonstrated no significant variations in psychological, environmental, and social relationship domains than those with stage A^19^. The study highlights the complex nature of mental health issues in CHD patients, emphasizing the necessity for targeted interventions to boost resilience and alleviate anxiety and depression.

A better QOL among patients with CHD than healthy control could be explained by a stronger sense of coherence in patients with CHD^26,27^. A sense of coherence is a measure of psychological resilience. The development of the sense of coherence is strengthened by growing up with CHD and the stressors associated with it^26^. This explains why individuals do well, despite the adversity of a chronic condition^28^. In our analysis, only one study reported the correlation between QOL and the sense of coherence of CHD patients; there was a significant positive correlation^16^. Better QOL and longer life span of CHD patients can be achieved by early recognition and urgent care^29^.

Regarding anatomical and physiological classification, the study reveals that anatomical classifications do not significantly impact QOL in CHD patients, highlighting the complexity of factors affecting well-being beyond anatomical considerations. However, in terms of physiological classification, higher stages of CHD are associated with lower physical domain scores, suggesting the physiological dimension may impact QOL.^19^. This suggests that the physiological classification, diagnosis, and stage of the disease can affect the QOL of patients with CHD.

Inconsistent findings were observed regarding the correlations between QOL and each disease severity and the severity of residual lesions^25^. It was reported that QOL is unrelated to subtypes of CHD and cyanosis^25^, whereas one study in our analysis showed a significant correlation between QOL and cyanotic heart disease^18^. It was reported that minor heart defects may not affect the QOL of the patient and even may not require any intervention. On the other hand, severe defects would require extensive medical support^2,6,30^. These observations emphasize the importance of considering disease severity as a key factor in understanding and addressing the diverse subtypes of patients with CHD and their overall QOL.

Determinants of QOL varied in different studies and included factors such as education, functional status, employment status, physical limitations, and personality type^25^. On the other hand, inconsistent findings were observed regarding the correlations between QOL and each age, gender, and medication^31^.

In our analysis, the female gender was associated with a lower score of physical function^20^, high education was a protective factor against poor QOL^18^, and one study showed that poor health-related QOL was associated with young age^14^, and lower QOL was more common among patients younger than 30 years. Also, unemployment and unstable employment had a four-fold risk for poor QOL^17^, and this was in agreement with the previous statement^25^.

Two systematic reviews on QOL among CHD adult patients study found heterogeneous populations, designs, disease severity classification, and QOL measurements in the included studies, resulting in variations and inconsistency in findings^9,32^. These limitations persist, even after recent studies were included, despite the inclusion of recent studies published after the previous systematic review.^9^.

The meta-analysis and systematic reviews on QOL among CHD patients illustrate the growth in evidence, but the findings were not always consistent^25^. It has been shown that the tools used to assess QOL determined the study’s findings. Hence, the research methodology that was used shaped the findings in each study which hampered the ability to draw firm conclusions. When certain instruments were used, CHD patients were found to report worse QOL than healthy peers. On the other hand, when other tools were used, patients reported the same level of QOL as healthy individuals^25^.

### Limitations

A major limitation of this study is its dependence on only two databases for data collection. Although these databases were inclusive, additional research databases could have provided a more comprehensive pool of findings and enhanced the generalizability of the study’s findings. Future research should consider including a wider range of databases to ensure a more diverse and representative sample, ultimately strengthening the validity of the study’s results.

Furthermore, the inclusion of studies published between 2020 and 2022 is acknowledged as a limitation. However, this timeframe was intentionally chosen to provide recent and realistic information that correlates with the current QOL determinants, as societal and environmental factors can change over time. Additionally, future research should also explore potential confounding variables that may influence the correlation between the identified determinants and quality of life.

The small sample size represents another limitation, preventing a comprehensive analysis. While it may be true that there is limited literature on QOL assessment in adult patients with CHD, it remains essential to review and analyze more studies to gain a better understanding of their potential effects and limitations.

This timeframe was chosen with the aim of providing current and accurate data that is consistent with the present determining factors of quality of life, as societal and environmental factors can change over time. Additionally, future research should also examine any potential confounding variables that could affect the relationship between the identified factors and quality of life.

## Conclusion

This study analyzes the QOL of adult patients with CHD, revealing that they have a lower QOL and health status compared to healthy individuals. The impact extends beyond physical dimensions, affecting psychological and social well-being. Patients with specific CHD diagnoses showed better QOL, physical functioning, and mental health, while those with cyanotic heart disease or Eisenmenger syndrome experienced poorer outcomes. Physiological classification revealed lower physical domain scores for higher stages of CHD. The study emphasizes the need for holistic care approaches, including psychosocial support, patient education, and specialized care.

Moreover, other risk factors for poor QOL included age, depression, anxiety, employment status, female gender, and poverty. However, precise results are required. Further studies focusing on specific diagnoses and specific dimensions of QOL.

## Ethical approval

Not available.

## Consent

Not available.

## Sources of funding

Not available.

## Author contribution

Conception and design of the study were done by A.M.A.M., S.H.M.K. Data extraction was done by A.M.A.M., S.H.M.K. Drafting and writing the manuscript was done by A.M.A.M., S.H.M.K. Revising the manuscript critically for important intellectual content was done by A.M.A.M., S.H.M.K. All authors approved the final manuscript.

## Conflicts of interest disclosure

Authors have no conflict of interest to declare.

## Research registration unique identifying number (UIN)

1. Name of the registry: Not applicable.
2. Unique Identifying number or registration ID: Not applicable.
3. Hyperlink to your specific registration (must be publicly accessible and will be checked): Not applicable.

## Guarantor

Ahmdelmukashfi M.Elmustfa A.Azem Mahmod.

## Availability of data and materials

The data of this study are available from the corresponding author upon reasonable request.

## Provenance and peer review

Not commissioned, externally peer-reviewed.
